# The mechanism of mitochondrial metabolic gene *PMAIP1* involved in Alzheimer's disease process based on bioinformatics analysis and experimental validation

**DOI:** 10.1016/j.clinsp.2024.100373

**Published:** 2024-04-30

**Authors:** Yingchun Ling, Lingmin Hu, Jie Chen, Mingyong Zhao, Xinyang Dai

**Affiliations:** a Department of Clinical Laboratory, Shaoxing Seventh People's Hospital, Shaoxing, Zhejiang, China; b Department of Geriatrics, Shaoxing Seventh People's Hospital, Shaoxing, Zhejiang, China

**Keywords:** Alzheimer's disease, Apoptosis, Biomarkers, Mitochondrial Metabolism

## Abstract

**Objectives:**

This study explored novel biomarkers that can affect the diagnosis and treatment in Alzheimer's Disease (AD) related to mitochondrial metabolism.

**Methods:**

The authors obtained the brain tissue datasets for AD from the Gene Expression Omnibus (GEO) and downloaded the mitochondrial metabolism-related genes set from MitoCarta 3.0 for analysis. Differentially Expressed Genes (DEGs) were screened using the “limma” R package, and the biological functions and pathways were investigated by Gene Ontology (GO) and Kyoto Encyclopedia of Genes and Genomes (KEGG) analyses. The LASSO algorithm was used to identify the candidate center genes and validated in the GSE97760 dataset. *PMAIP1* with the highest diagnostic value was selected and its effect on the occurrence of AD by biological experiments.

**Results:**

A sum of 364 DEGs and 50 hub genes were ascertained. GO and KEGG enrichment analysis demonstrated that DEGs were preponderantly associated with cell metabolism and apoptosis. Five genes most associated with AD as candidate central genes by LASSO algorithm analysis. Then, the expression level and specificity of candidate central genes were verified by GSE97760 dataset, which confirmed that *PMAIP1* had a high diagnostic value. Finally, the regulatory effects of *PMAIP1* on apoptosis and mitochondrial function were detected by siRNA, flow cytometry and Western blot. siRNA-*PMAIP1* can alleviate mitochondrial dysfunction and inhibit cell apoptosis.

**Conclusion:**

This study identified biomarkers related to mitochondrial metabolism in AD and provided a theoretical basis for the diagnosis of AD. *PMAIP1* was a potential candidate gene that may affect mitochondrial function in Hippocampal neuronal cells, and its mechanism deserves further study.

## Highlights

• PMAIP1 may be a potential biomarker for Alzheimer's Disease.
• PMAIP1 has a certain pro-apoptotic effect.
• PMAIP1 is participate in hippocampal neuron damage by regulating mitochondrial function.

## Introduction

Alzheimer's Disease (AD) is the most common slowly progressive neurodegenerative disease. According to the World Alzheimer's Report 2018, as the population ages, the number of people with AD will increase to 152 million by 2050.^1^ Particularly, age-specific global prevalence in women was 1.17 times larger than in men and the age-standardized mortality rate of women was also higher than men.^2^ In addition, death tolls with AD increased 145% from 2000 to 2019 and AD became the fifth-largest cause of death in American old people.^3^ Notably, caregivers are likely to face increased mental strain and negative emotional impacts. The social and family responsibilities associated with caring for individuals with AD could become overwhelmingly burdensome and difficult to sustain.^4^ However, the cellular and molecular mechanisms that contribute to the pathogenesis of AD have not been fully elucidated.

The main features of AD are neuritic plaques and neurofibrillary tangles as a result of Amyloid-Beta peptide's (Aβ) accumulation in the most affected area of the brain, the medial temporal lobe and neocortical structures.^5^ Neurons possess unique polarized structures and have high energy demands, relying heavily on mitochondrial function to provide the energy required for sustaining neuronal activity and survival.^6^ Key early pathological events in AD include mitochondrial dysfunction, leading to the overproduction and accumulation of Reactive Oxygen Species (ROS), which impair neuronal function long before the onset of AD symptoms and pathological hallmarks.^7^^,^^8^ Research indicates that FRMD6 can mitigate the toxic damage caused by Aβ by improving mitochondrial function and structure in hippocampal neurons.^9^ Therefore, a clear understanding of the involvement of mitochondria in hippocampal neuronal damage caused by AD is crucial for the treatment of AD.

In recent years, integrated bioinformatics analysis has been used to identify new genes associated with various diseases, which may serve as biomarkers for diagnosis and prognosis.^10^^,^^11^ However, the common diagnosis and interlinked genes in Mitochondrial Metabolism and AD are unclear. In this study, the authors screened Differentially Expressed Genes(DEGs) between AD patients and control samples using the “Limma” R package and constructed a Protein-Protein Interaction network (PPI) using Cytoscape software. Gene Ontology (GO) and Kyoto Encyclopedia of Genes and Genomes (KEGG) pathway enrichment analyses were then performed to explore the biological pathways enriched in DEGs. The DEGs were crossed with the mitochondrial metabolism-related genes to obtain the differentially expressed metabolism-related genes, and the candidate genes were screened by LASSO regression analysis. The Receiver Operating Characteristic (ROC) curve was drawn to explore the sensitivity and specificity of the candidate genes for AD. Finally, the authors determined that *PMAIP1* has a high diagnostic value. In vitro experiments further showed that *PMAIP1* may be involved in Aβ-induced hippocampal neuron injury by regulating mitochondrial function. Therefore, this study may provide preliminary insights into the role of *PMAIP1* in AD cells and facilitate further investigation the targeted therapy for AD patients.

## Materials and methods

### Microarray data processing

Three microarray datasets (GSE5281, GSE28146, and GSE97760) of AD were downloaded from Gene Expression Omnibus (GEO) (Home - GEO – NCBI (nih.gov)). Ten AD patients and 13 control samples were selected from GSE5281 belonging to the hippocampus series. GSE28146 contains 22 AD patients and 8 control samples. They are all based on the GPL570 (Affymetrix Human Genome U133 Plus 2.0 Array) platform. In order to further verify the reliability of the results, the dataset GSE97760 was used as the validation set, which included 9 AD patients and 10 control samples, based on the platform of GPL16699 (Agilent-039494 SurePrint G3 Human GE v2 8 × 60 K Microarray 039381). In addition, this study is a diagnostic and prognostic study, following the Standards for Reporting of Diagnostic Accuracy (STARD) guidelines.

### Identification of DEGs

The “limma” package in R software (Version 4.3.1) was utilized to screen DEGs between the AD patients and control samples. An adjusted p-value of < 0.05 and log2 |Fold Change|>1.0 were considered statistically significant. The “ggplot2” package in R was used to plot the volcano map in the two groups.

### Functional enrichment analysis

To further visualize the biological function of DEGs, GO and KEGG enrichment analyses were identified in the Comparative Toxicogenomics Database (The Comparative Toxicogenomics Database | CTD (ctdbase.org)). A p-value of less than 0.05 was identified as a significant term. The “ggpubr” and “ggplot2” package in R was used to visualize the results.

### PPI networks

To identify and assess protein functional relationships and PPI networks for differentially expressed mRNAs, the authors utilized the Search Tool for the Retrieval of Interacting Genes (STRING: functional protein association networks (string-db.org)). The results of the STRING analysis were then imported into Cytoscape, which was used to select the key nodes with the strongest connectivity to visualize the molecular interaction network. The nodes with the most interactions with neighboring nodes were considered as the key nodes. Evaluate the importance of each node through CytoHubba and select the top 50 nodes.

### Identification of DEGs related to mitochondrial metabolism

MitoCarta3.0 is an inventory of 1136 human and 1140 mouse genes encoding proteins with strong support of mitochondrial localization, now with sub-mitochondrial compartment and pathway annotations.^12^ The mitochondrial metabolism-related genes were downloaded from the MitoCarta3.0 (MitoCarta3.0: An Inventory of Mammalian Mitochondrial Proteins and Pathways | Broad Institute), including the 1136 mitochondrial human genes. These genes were compared to DEGs. The overlapping genes were described by the Venn diagram.

### Machine learning to screen candidate genes

Least Absolute Shrinkage and Selection Operator (LASSO) is a regression analysis method for variable selection and regularization, which can improve the prediction accuracy and interpretability of statistical models.^13^ The R package “glmnet” was used for the LASSO analysis to identify the most valuable predictive genes. The genes and their coefficients were determined by the best penalty parameter λ associated with the smallest 10-fold cross-validation.

### Evaluation of candidate gene diagnostic value

The Area Under the Curve (AUC) from a ROC curve analysis was calculated to test the diagnostic performance of each candidate gene. It was verified in GSE97760. The R package “pROC” was used for drawing ROC curves.

### Cell culture and preparation of Aβ1–42

HT-22 hippocampal cells (HT-22 cells) were obtained from the Chinese Academy of Sciences (Shanghai, China), and the cells were cultured in Dulbecco's Modified Eagle's Medium (DMEM) with 10% Fetal Bovine Serum (FBS), 1% penicillin and 1% streptomycin in a humidified 5% CO_2_ atmosphere at 37°C. Cells were treated with 5 μM, 10 μM, and 20 μM Aβ1-42 oligomer, respectively. Aβ1-42 oligomer preparation method: Aβ1-42 (Abcam, USA) dissolved in DMSO, ultrasonic 5 min in the cold bath, then immediately stored at -80°C. The Aβ1-42/DMSO solution was diluted with serum-free DMEM to a final concentration of 100 μM and stored at 37°C.^14^

### Small interfering RNA transfection

The target sequence of Phorbol-12-Myristate-13-Acetate-induced Protein 1 (*PMAIP1*) is 5′-GGAAGUCGAGUGUGCUACU-3′. The transfection of siRNA followed the manufacturer's protocol of X-tremeGENE Transfection Reagent (Roche Molecular Biochemicals, Mannheim, Germany). Cells were vaccinated in 6-well plates, and transfected with control or target siRNA on the next day. Cells were treated with indicated Aβ1-42 oligomer (20 μM) for 48h and harvested for subsequent experimental analysis.

### Cell viability assay

HT-22 cells were seeded at a density of 3 × 10^3^ cells/in 96-well microtiter plates. The next day, the cells were treated with the presence and absence of 5, 10, 20 μM Aβ1-42 oligomer for 48h. The viability of HT-22 cells was detected using a Cell Counting Kit-8 (CCK-8) assay according to the manufacturer's instructions. The absorbance at 450 nm was measured by a microplate Reader (Bio-Rad, La Jolla, CA, USA). Similarly, using the CCK-8 assay, the viability of *PMAIP1*-siRNA cells was measured at a concentration of Aβ1-42 oligomers of 20 μM.

### Western blot analysis

HT-22 cells were added to RIPA lysis buffer on ice and lysed for 10 minutes. The lysate was then centrifuged at 12000 rpm for 10 minutes at 4°C, and the supernatant was collected to obtain the total protein solution. The protein concentration was determined with the Enhanced BCA Protein Assay Kit (Beyotime Biotechnology, China). The samples were separated by 10% SDS-PAGE (Bio-Rad, CA) and transferred to polyvinylidene fluoride membranes (Millipore, USA). Then the blots were incubated at 4°C overnight with the primary antibodies: anti-*PMAIP1*, anti-BCL2, anti-Bax, and anti-caspase-3. Subsequently, the membranes were washed three times with 1 × TBST and incubated with secondary antibodies for 2h at room temperature. Next, the membranes were scanned using the Odyssey® CLx Imaging System (LI-COR Biosciences, United States) and the density of the bands was determined using ImageJ software.

### Flow cytometric cell death analysis

Apoptosis was assessed using the Annexin V-FITC/PI apoptosis detection kit (Nanjing, China) according to the manufacturer's instructions. 2 × 10^6^ cells were harvested and washed twice with pre-cold PBS and then resuspended in 500 μL binding buffer. 5 μL annexin V-FITC and 5 μL Propidium Iodide were added to each sample and then incubated for 10 minutes at room temperature in the dark. Analysis was performed by FACScan flow cytometer (Becton Dickinson, CA).

### ROS assay

To identify the ROS production, a fluorescenceprobe 2, 7-dichlorofluorescin diacetate kit (DCFH-DA, Sigma) was used. HT-22 cells were washed with PBS and incubated using DCFH-DA at 37°C without light for 30 min. Then washed cells three times with PBS and the fluor­escence intensity was detected by using a fluorescence microscope (Thermo Fisher Scientific, Waltham, MA, USA).

### Evaluation of mitochondrial membrane potential (MMP)

Cationic dye 5, 5ʹ, 6, 6ʹ-tetrachloro-1, 1ʹ, 3, 3-tetraethylbenzimidazolyl-carbocyanine iodide (JC-1, Sigma-Aldrich, MO, USA) staining was conducted to assess MMP. Red fluorescence represented a potential-dependent aggregation in the mitochondria, reflecting ΔΨm. The green emission of the dye represented the monomeric form of JC-1. The wavelengths of excitation and emission were 514 nm and 529 nm for the detection of the monomeric form of JC-1, while 585 nm and 590 nm were used to detect aggregation of JC-1.

### Detection of malondialdehyde (MDA), Superoxyde Dismutase (SOD) level

The MDA and SOD contents in the HT-22 cells were assessed using a Lipid Peroxidation MDA Assay Kit and SOD Assay Kit (Beyotime Biotechnology, China). The test was performed according to the manufacturer's instructions, and the absorbance at 532 nm was recorded using a microplate reader (Benchmark; Bio-RadLaboratories, Inc.).

### Statistical analysis

The differences between groups were examined using one-way ANOVA followed by the Bonferroni post hoc-test. For all analyses, p-values < 0.05 were considered statistically significant.

## Results

### Identification of DEGs

**Fig. 1:**
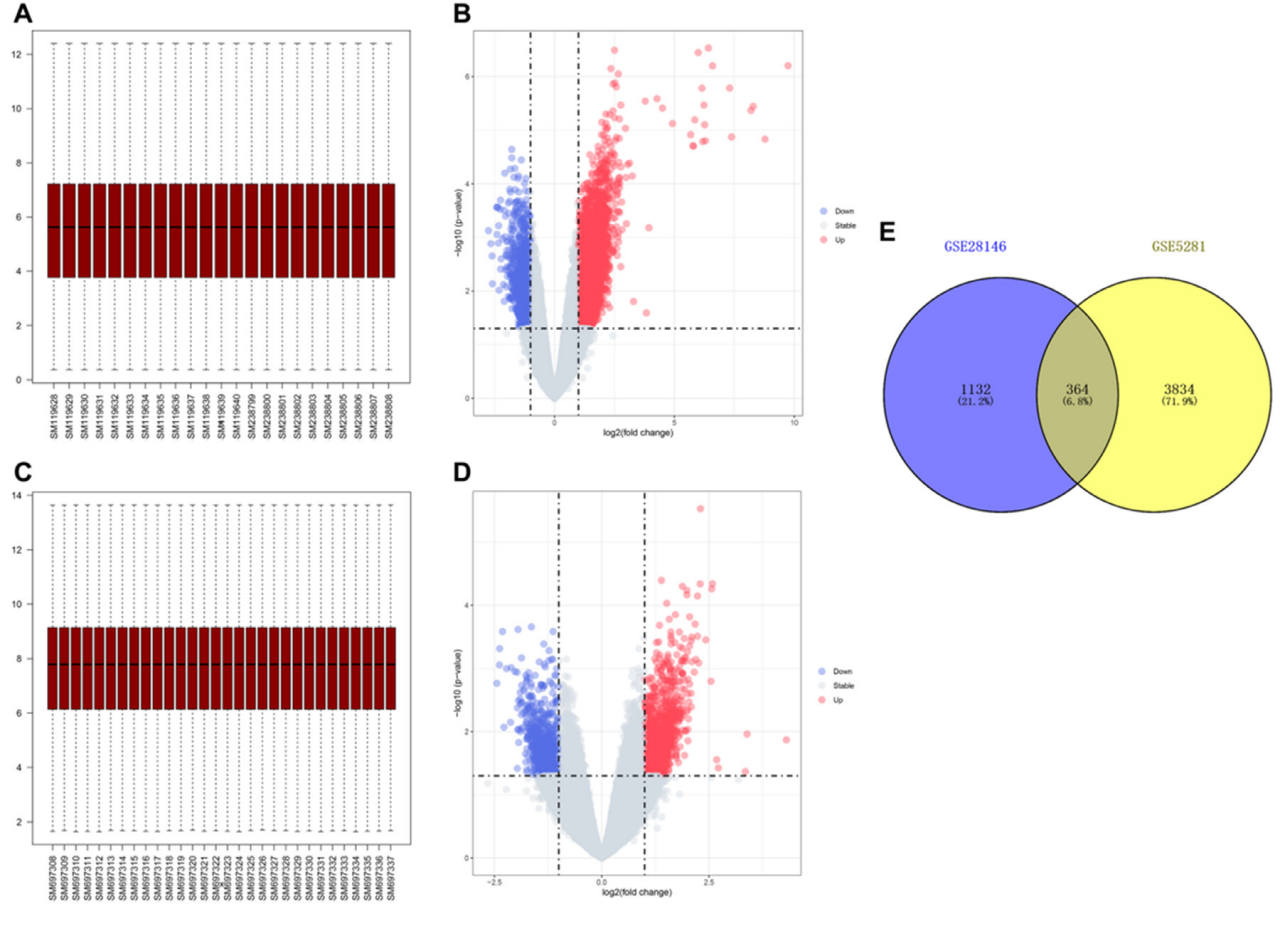
**Data preprocessing and identification of DEGs**. (A) Boxplot of transcriptome data of GSE5281. (B) Volcano plot of DEGs in GSE5281. The cut-off criteria were |log2Fc|>1 and p < 0.05. The red dots represent the up-regulated genes, and the blue dots denote the down-regulated genes. The grey dots indicate the genes with |log2Fc|<1 and/or p > 0.05. (C) Boxplot of transcriptome data of GSE28146. (D) Volcano plot of DEGs in GSE5281. (E) Venn diagram showing the numbers of overlapped DEGs between GSE5281 and GSE28146.

To investigate the differences in gene expression between AD samples and control samples, the authors used the GSE5281 dataset which contained 10 AD samples, and 13 control samples to identify 4198 DEGs (Fig. 1 A‒B). At the same time, another sample dataset GSE28146 which included 22 AD samples and 8 control samples was used to verify 1496 DEGs (Fig. 1 C‒D). In addition, a Venn diagram analysis was performed to evaluate the 364 common DEGs (Fig. 1E).

### Functional enrichment analysis of DEGs

**Fig. 2:**
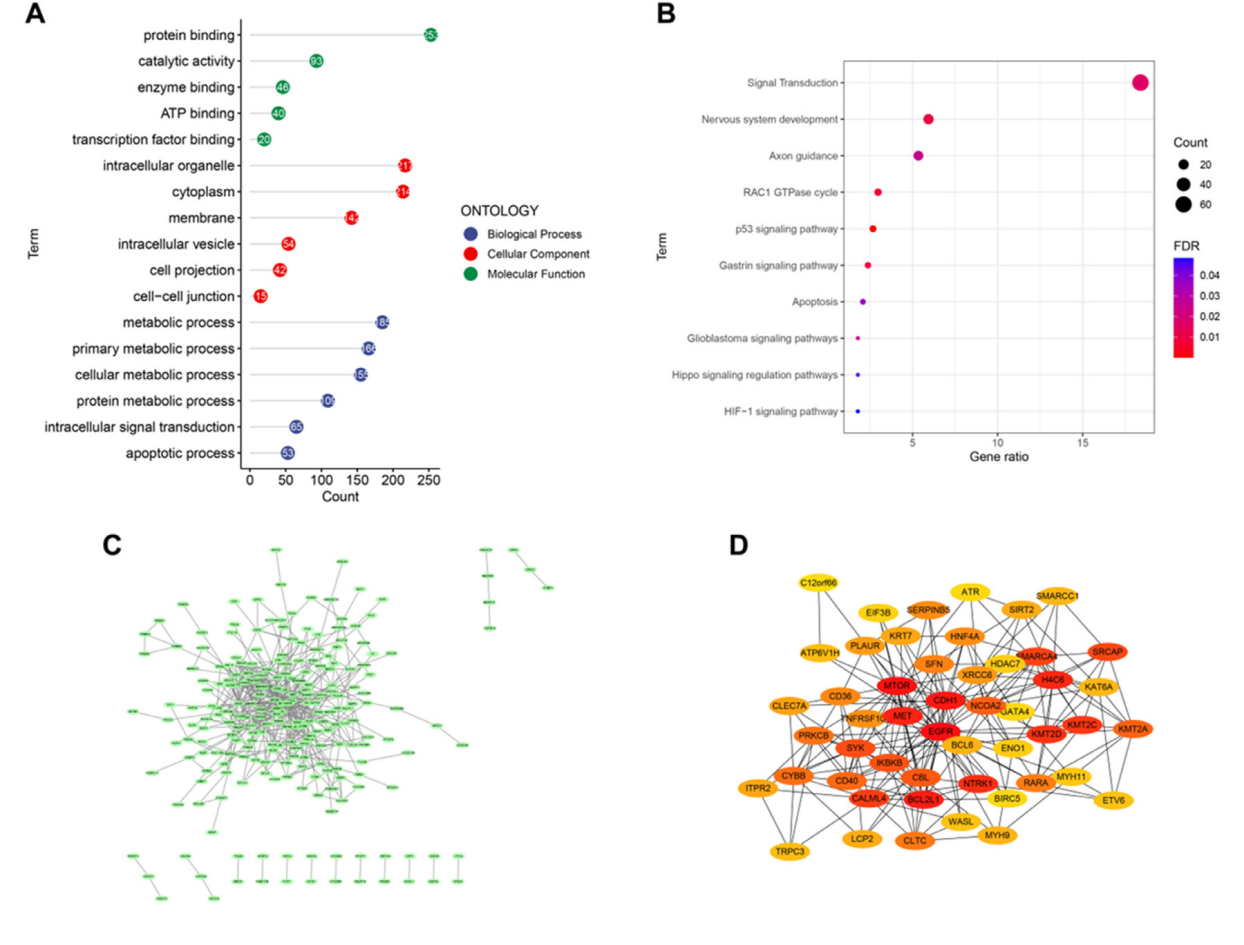
**Functional enrichment analysis and PPI network.** (A) GO functional analysis showing enrichment of DEGs. (B) KEGG pathway enrichment analysis of DEGs. (C) PPI network of DEGs were analyzed using Cytoscape software. (D) PPI network for the top 50 genes.

GO analysis was conducted to obtain the biological functions of 364 DEGs to understand which signaling pathways might serve an important role in AD. The significant terms of biological processes were principally associated with the metabolic, such as cellular metabolic, and protein metabolic. The pathways enriched by molecular function were principally associated with protein binding, catalytic activity, and transcription factor binding. The analysis of cellular components indicated that DEGs were significantly enriched in cytoplasm, membrane, intracellular vesicle (Fig. 2A). The KEGG analysis showed that these genes were enriched in Apoptosis and Hippo signaling regulation pathways (Fig. 2B).

### PPI network establishment and identification of hub genes

To explore the relationship between these DEGs and to identify hub genes, a PPI network of DEGs was constructed using the STRING online database and visualized using Cytoscape. There were 229 nodes and 542 edges in the PPI network (Fig. 2 C‒D).

### Identification of DEGs related to mitochondrial metabolism

**Fig. 3:**
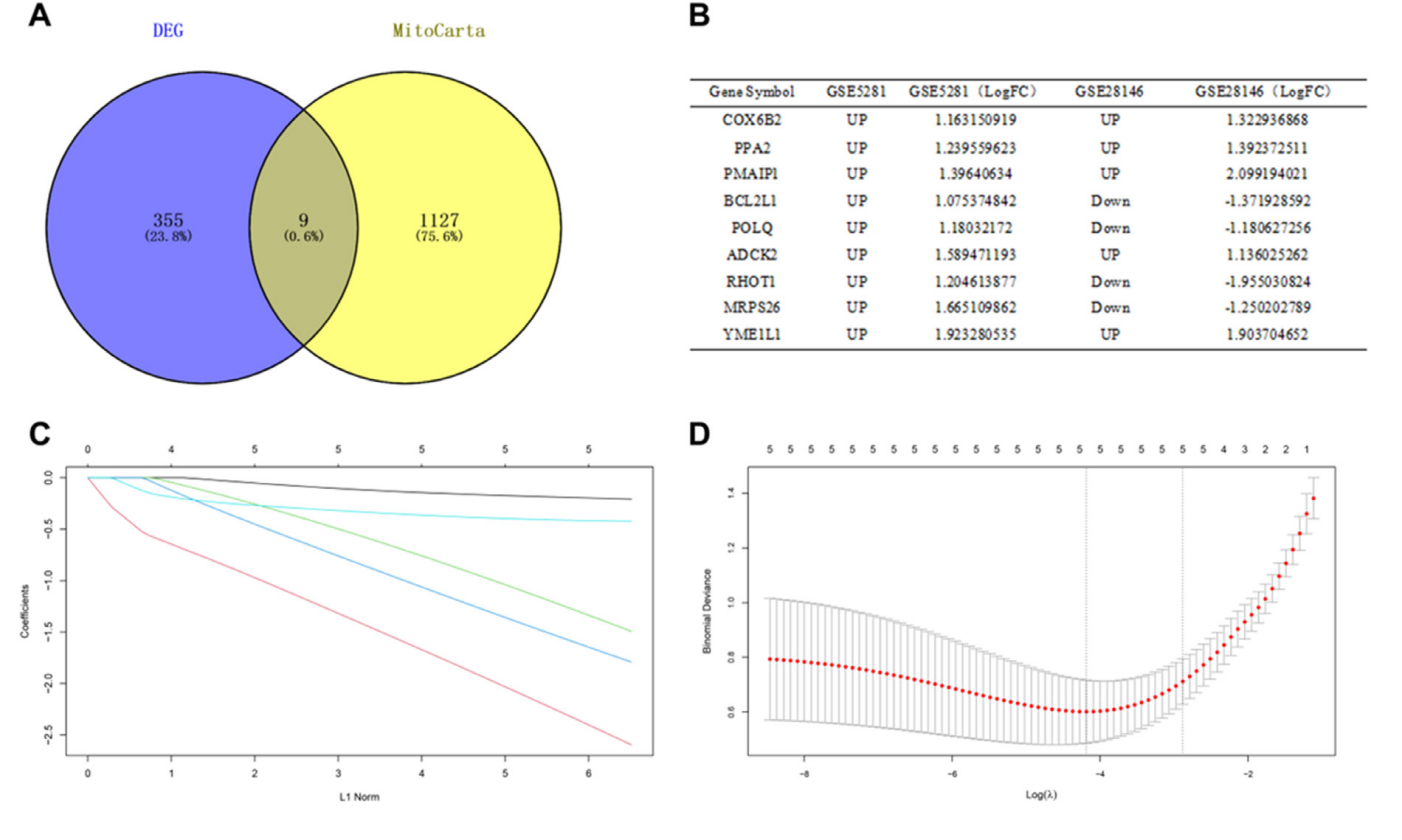
**Identification of candidate central genes.** (A) Venn diagram showing the numbers of overlapped genes between DEG s and MitoCarta. (B) Expression trends of overlapping genes in GSE5281 and GSE28146 datasets. (C) LASSO coefficient profiles of candidate genes. (D) Cross-validation to select the optimal tuning parameter log (λ) in LASSO regression analysis.

The 1136 mitochondrial metabolism-related genes were downloaded from MitoCarta3.0. The authors overlapped the 364 DEGs from GSE5281 and GSE28146 with mitochondrial metabolism-related genes, 9 overlapped genes were obtained (Fig. 3A). Among them, the trends of 5 genes (*COX6B2, PPA2, PMAIP1, ADCK2, YME1L1*) expressions were same in GSE5281 and GSE28146 datasets (Fig. 3B).

### Identification of candidate central genes using machine learning

**Fig. 4:**
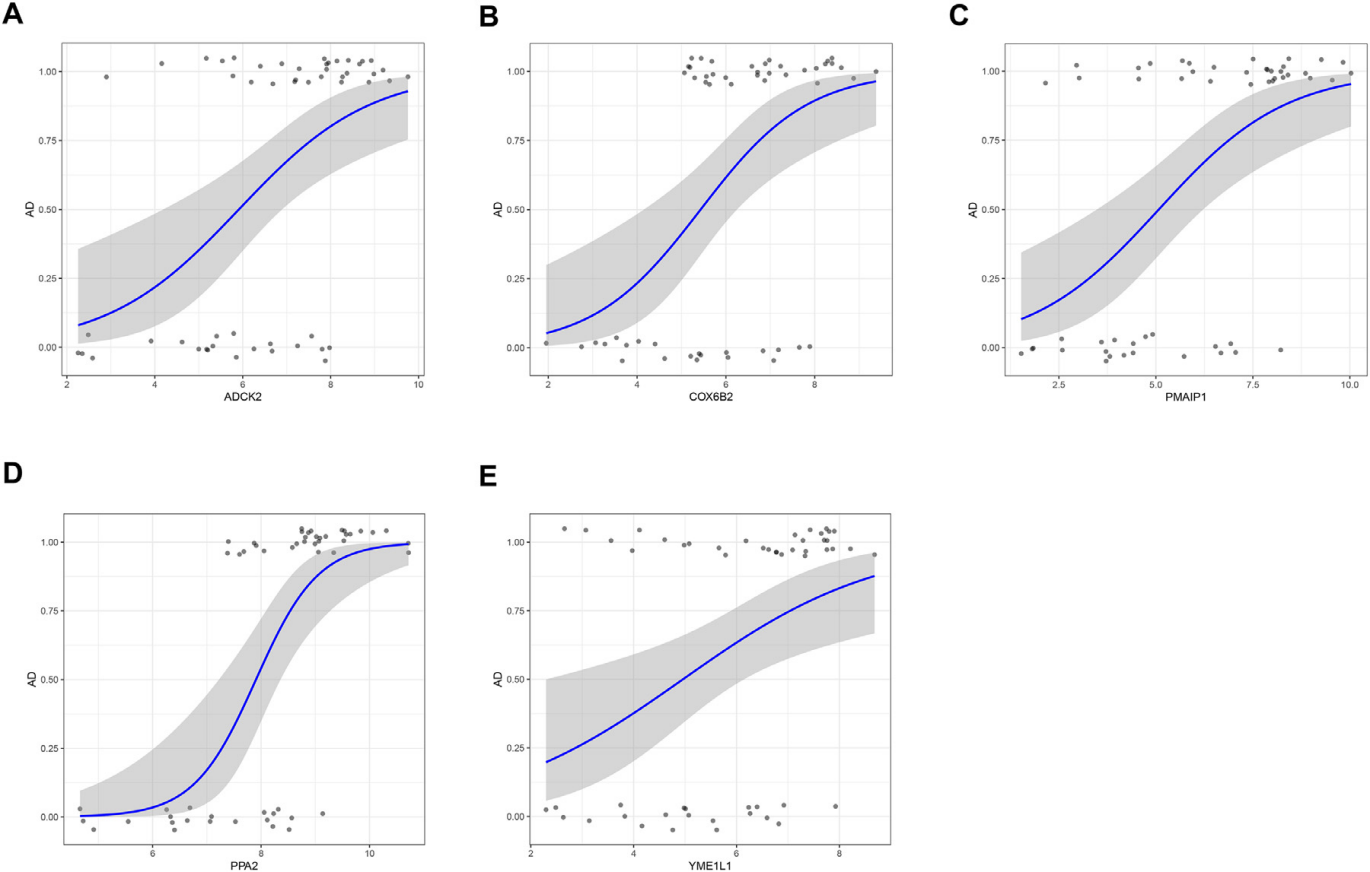
The relationship between 5 genes expression and AD using the method of binomial logistic regression for generalized linear models. (A) *ADCK2* (B) *COX6B2* (C) *PMAIP1* (D) *PPA2* (E) *YME1L1*.

**Fig. 5:**
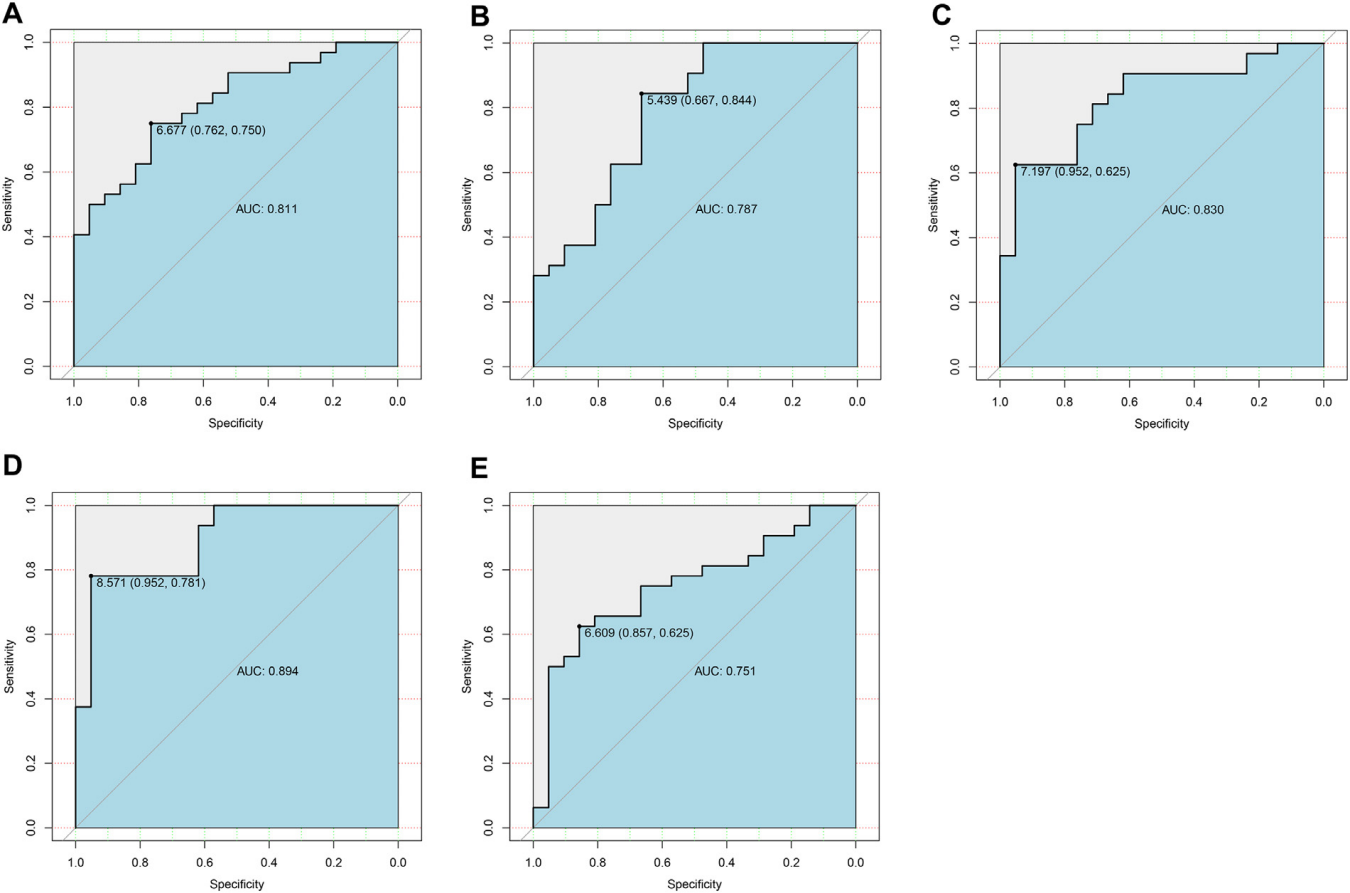
**The AUCs of 5 candidate central genes**. (A) *ADCK2* (B) *COX6B2* (C) *PMAIP1* (D) *PPA2* (E) *YME1L1*.

Candidate genes were further screened based on LASSO regression and 10-fold cross-validation. All five genes were included as candidate genes, namely *COX6B2, PPA2, PMAIP1, ADCK2, YME1L1* (Fig. 3 C‒D)*.* The relationship between candidate gene expression and AD was then investigated by binomial logistic regression of generalized linear models. The results of this model suggest that the relationship is monotonic. Meanwhile, the risk of AD increases with increased candidate gene expression (Fig. 4 A‒E). The authors further evaluated the diagnostic values of these genes. The AUC values of ROC curves were 0.811 of *ADCK2*, 0.787 of *COX6B2*, 0.830 of *PMAIP1*, 0.894 of *PPA2*, 0.751 of *YME1L1*. The authors found that they all had high accuracy with AUC > 0.75, revealing the predictive efficacy of all 5 gene signatures (Fig. 5 A‒E).

### External dataset validation

**Fig. 6:**
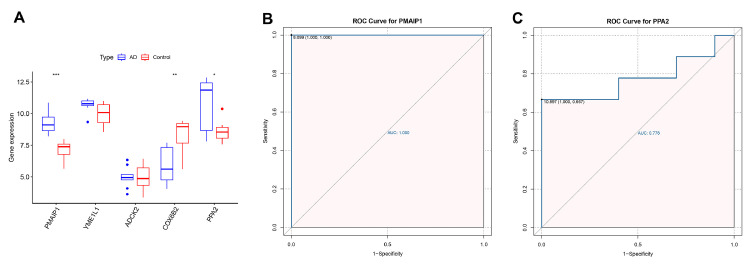
**External dataset validation.** (A) The expression patterns of 5 genes from the GSE97760 dataset. * p < 0.05, ** p < 0.01, *** p < 0.001. (B) The AUC of *PMAIP1* in the GSE97760 dataset. (C) The AUC of *PPA2* in the GSE97760 dataset.

The authors further plotted box plots designed to clearly demonstrate the expression variations of the 5 candidate central genes in the GSE97760 dataset. The results showed that *PMAIP1, PPA2, YMEL1, ADCK2* were elevated in the AD patients, while the *COX6B2* were overexpressed in the control samples. However, only *PMAIP1* and *PPA2* showed significant differences in expression between AD patients and control samples (Fig. 6A). In addition, the authors further mapped the ROC curves of these two genes. As shown in ROC analysis, the AUC values of *PMAIP1* and *PPA2* reached 1.00 and 0.778 (Fig. 6 B‒C). These results indicate that *PMAIP1* is highly correlated with the sensitivity and specificity of AD patients.

### PMAIP1 expression increased in Aβ-induced HT-22 cells

**Fig. 7:**
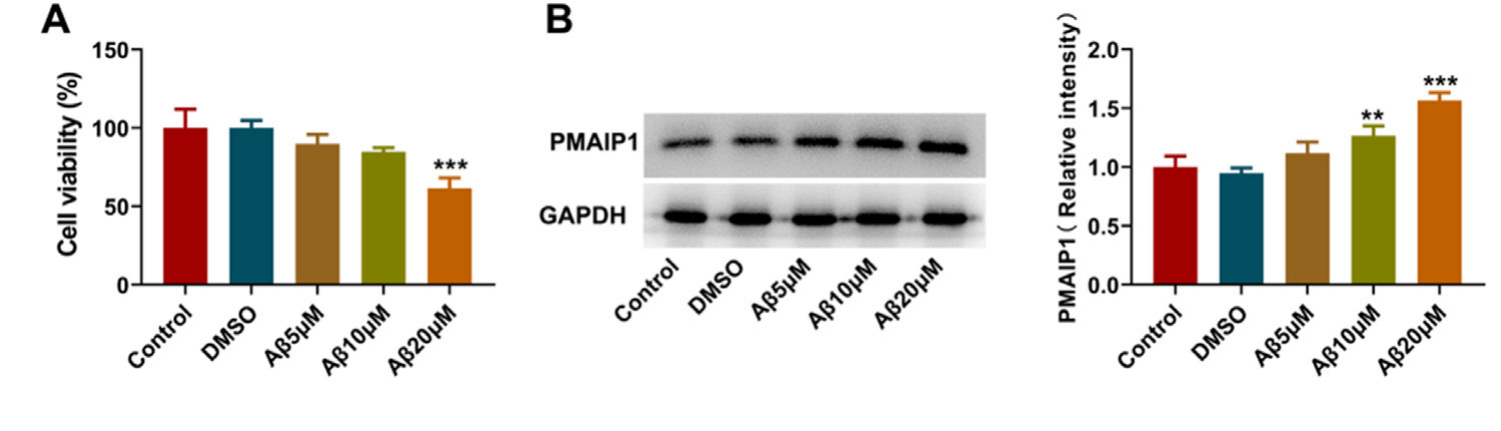
***PMAIP1* expression in Aβ-induced HT-22 cells**. (A) The viability of HT-22 cells treated with A-β1-42 (5, 10 and 20 µM) was measured by CCK-8 assay. (B) Protein levels of *PMAIP1* measured by western blot assay. ** p < 0.01, *** p < 0.001 versus control.

To investigate the expression of *PMAIP1* in hippocampal neurons in AD, the authors first used Aβ oligomer to construct a cell model of AD. CCK8 assay showed that 20 μM Aβ-induced HT-22 cells had the lowest cell viability (Fig. 7A). Western blotting was used to detect the expression of *PMAIP1* protein. The expression level of *PMAIP1* was significantly increased in the Aβ-induced HT-22 cells compared with the control group (Fig. 7B). In subsequent experiments, Aβ (20 μM) was selected to construct the AD cell model.

### siRNA-PMAIP1 inhibited the apoptosis of Aβ-induced HT-22 cells

**Fig. 8:**
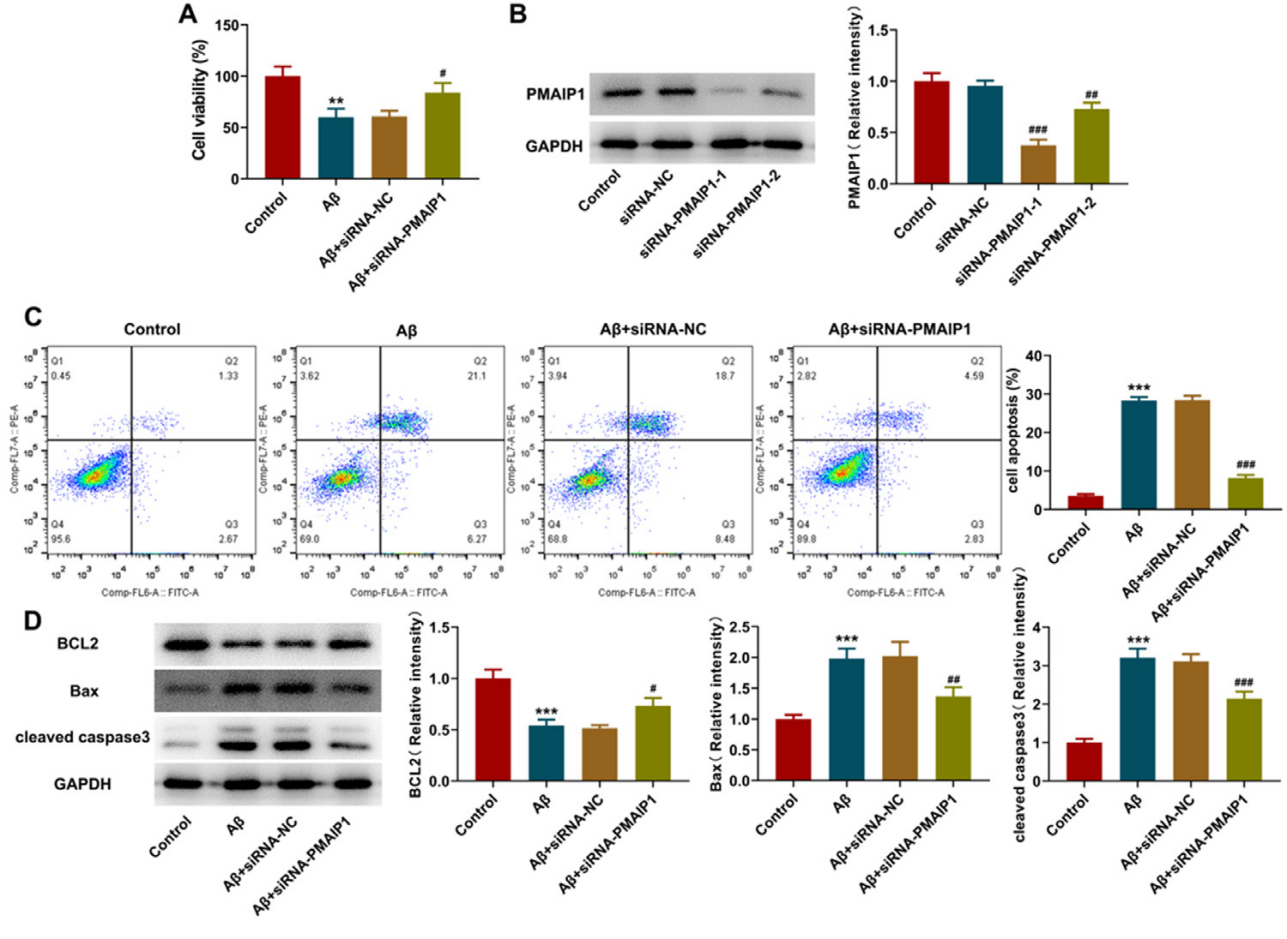
**Effect of siRNA-*PMAIP1* on Aβ-induced apoptosis in HT-22 cells.** (A) Protein levels of *PMAIP1* after small RNA interfering measured by western blot assay. (B) Cell viability was detected by CCK8 assay. (C) Apoptotic status of HT-22 cells was assayed by flow cytometry. (D) The expression levels of BCL2, Bax and cleaved caspase3 were measured by western blot. *** p < 0.001 versus control. ^#^ p < 0.5, ^##^ p < 0.01, ^###^ p < 0.001 versus siRNA-NC.

After small interfering RNA transfection, a western blot was used to detect the interference level of *PMAIP1*. Both siRNA sequences caused a significant reduction of *PMAIP1* after transfection, and the transfection efficiency of siRNA-*PMAIP1*-1 was higher than that of siRNA-*PMAIP1*-2 (Fig. 8A). Therefore, siRNA-*PMAIP1*-1 sequence was selected as the final experimental sequence for subsequent research in this study. Compared with the siRNA-NC group, the cell viability was increased, and the apoptosis rate was significantly decreased after siRNA-*PMAIP1* transfection (Fig. 8 B‒C). At the same time, the expression levels of Bax and cleaved caspase3 were decreased, and the expression level of BCL2 was increased, suggesting that *PMAIP1* promoted the apoptosis of Aβ-induced HT-22 cells (Fig. 8D).

### siRNA-PMAIP1 reduces the mitochondrial damage in Aβ-induced HT-22 cells

**Fig. 9:**
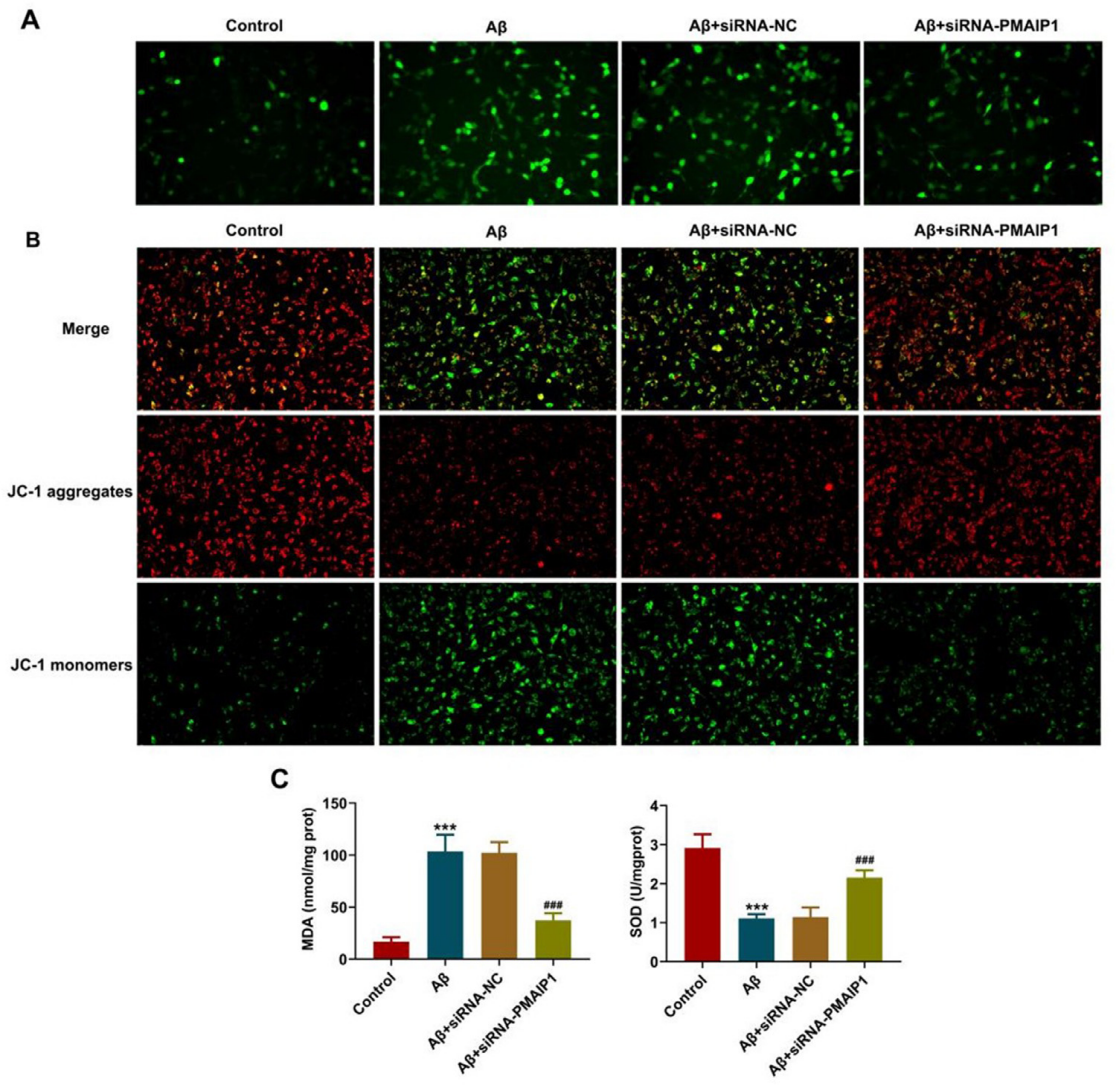
**Effect of siRNA-*PMAIP1* on Aβ-induced mitochondrial function in HT-22 cells.** (A) ROS level was detected by DCFH-DA staining. Original magnification: × 200. (B) MMP was identified by JC-1 staining. JC-1 exists in both aggregates and monomers states. Green fluorescence indicates that JC-1 exists as a monomer at low concentrations, and red fluorescence indicates that JC-1 exists as an aggregate at high concentrations. Original magnification: × 200. (C) The activity of MDA and SOD. *** p < 0.001 versus control. ### p < 0.001 versus siRNA-NC.

It has been reported that the mitochondrial targeting domain contained in *PMAIP1* is capable of inducing mitochondrial swelling and Mitochondrial Permeability Transition Pore (MPTP) opening and oxidative stress generation.^15^ With this in mind, the authors evaluated mitochondria injury in HT-22 cells. ROS production was significantly increased after treatment with Aβ, but this effect was reversed by siRNA-*PMAIP1* treatment (Fig. 9A). As shown in the figure, the aggregated JC-1 in the mitochondria of normal hippocampal neurons showed red fluorescence, Aβ treatment significantly reduced the aggregated JC-1 and increased the monomeric JC-1, but siRNA-*PMAIP1* reduced the dissipation of ΔΨm, showing more red fluorescence and less green fluorescence (Fig. 9B). In addition, compared with siRNA-NC, the levels of MDA in siRNA-*PMAIP1* group was significantly reduced, while the levels of SOD in siRNA-*PMAIP1* group was significantly increased (Fig. 9C). These results indicate that mitochondrial dysfunction can be reduced after siRNA-*PMAIP1* interference.

## Discussion

Alzheimer's Disease (AD) is an age-related neurodegenerative disorder that leads to progressive cognitive decline and neuronal cell death.^16^ Cumulative studies of the cellular model,^17^ AD mouse model,^18^ and AD brain tissue^19^ have shown that Aβ is present in many forms in an AD-affected brain including monomers, toxic oligomeric intermediates, and fibrils. The soluble oligomeric form, particularly Aβ1-42, produces cytotoxic effects that initiate a cascade of events that contribute to the development of AD due to a higher propensity to aggregate.^20^^,^^21^ Meanwhile, Aβ-induced oxidative stress is a key factor in the formation of AD.^22^ Therefore, in this paper, the authors constructed a model of Aβ-induced damage to hippocampal neurons as a cellular model for this study according to the methods reported in the literature.^14^ Existing studies have also shown that there are not only in vitro models of AD but also in vivo models including Aβ1-42 intraventricular injection,^23^ APP/PS1 mice,^24^ scopolamine-induced^25^ and streptozotocin-induced^26^ AD animals. This is the limitation of this paper. The next step should be further validation by in vivo experiments to evaluate its impact on the prognosis, treatment, and diagnosis of AD.

Mitochondrial dysfunction plays a pivotal role in the pathogenesis of AD.^27^ Several studies have confirmed that impaired mitochondrial function underlies cognitive decline in aging and is one of the most notable hallmarks of AD.^28^^,^^29^ Recent studies suggest that neurons have particularly high and sustained energy requirements, rely heavily on mitochondrial ATP supply, and are particularly sensitive to mitochondrial dysfunction. Mitochondrial damage can cause energy crises, oxidative stress, and impaired cellular signaling, which have been linked to the pathogenesis of neurodegenerative diseases.^30^^,^^31^ In view of the above evidence, an in-depth analysis of mitochondrial-related molecular patterns is needed to provide a reliable research direction for future experimental studies and a theoretical basis for AD biomarkers to promote the diagnosis and treatment of AD.

In this study, the authors intersected the DEGs of the two datasets (GSE5281, GSE28146) to obtain 364 DEGs. Furthermore, 9 mitochondrial metabolism-related DEGs were obtained by intersections with genes related to mitochondrial metabolism. The authors compared the expression trends of these genes in the two datasets to obtain 5 genes. Through the analysis of the LASSO algorithm, the authors obtained 5 genes (*COX6B2, PPA2, PMAIP1, ADCK2, YME1L1*) most associated with AD as candidate central genes. The diagnostic value of these genes was determined by AUC values under the ROC curve. The authors found that all five genes have a high degree of accuracy. Finally, the expression level and specificity were verified by GSE97760 datasets, and 2 genes (*PPA2, PMAIP1*) were screened. *PMAIP1* with a higher diagnostic value was selected for biological experiment verification.

*PMAIP1* also known as *NOXA*, a member of the pro-apoptotic BH3-only protein family, is located at the outer mitochondrial membrane that promotes mitochondrial fragmentation and apoptosis. It enables the release of Bak/Bax, which enables it to bind to anti-apoptotic Mcl-1 and A1, thus possessing a pro-apoptotic function.^32^^,^^33^ Apoptosis is an important biological process in neurodegenerative disorders, while *PMAIP1* is an essential mediator of p53-dependent apoptosis, the deletion of it decreased the magnitude of apoptosis.^34^^,^^35^ *PMAIP1* overexpression was demonstrated to inhibit cell proliferation, while *PMAIP1* silencing promotes cell growth recovery.^36^ In addition, in Aβ-induced neurons, *PMAIP1* can promote the apoptosis of Aβ42-induced SH-SY5Y cells.^37^ In the present study, the expression level of *PMAIP1* was significantly increased in Aβ-induced HT-22 cells, while the cell activity was increased and the apoptosis rate was significantly decreased after siRNA-*PMAIP1* interference, indicating that *PMAIP1* has a certain pro-apoptotic effect, which is consistent with literature reports.

Mitochondria produce ROS, which are thought to increase intracellular oxidative stress and may lead to progressive cellular dysfunction that can lead to apoptosis. Early studies have shown that mitochondrial ROS are associated with the occurrence and poor prognosis of many diseases, so it is regarded as an important risk factor threatening human health.^38^ As reported by Childs et al., ROS production in mitochondria leads to the loss of MMP, and direct activation of MPTP.^39^ The end result of oxidative stress within mitochondria is the dissipation of MMP and the subsequent release of cytochrome c, which is also a critical event in neuronal degeneration.^40^ In the present study, ROS production was increased in Aβ-induced HT-22 cells, while it was reduced after siRNA-*PMAIP1* transfection, with increased levels of MMP and SOD and decreased levels of MDA. It is suggesting that *PMAIP1* is likely to participate in Aβ-induced hippocampal neuron damage by regulating mitochondrial function. Therefore, *PMAIP1* may be a potential biomarker for AD.

There were some limitations to this study. The authors used bioinformatic analysis to screen out molecular markers associated with mitochondrial metabolism in AD However, a prospective cohort is needed to further determine its diagnostic performance. In the future, after processing PMAIP1 interference, the authors will employ sequencing technology to analyze the genes and downstream signals regulated after PMAIP1 interference. In addition, the authors hope to complete it in further work to understand better the role of mitochondrial metabolism-related molecular mechanisms in the pathogenesis of AD.

## Conclusion

In Summary, the authors identified two key genes (*PMAIP1* and *PPA2*) that are closely associated with mitochondrial metabolism in AD and can differentiate AD patients from controls and are thus potential mitochondrial metabolism-related biomarkers for disease diagnosis and therapeutic monitoring. The present study provides a new idea for further understanding the role of mitochondrial metabolism in AD and its molecular mechanism and also provides a theoretical basis for increasing the diagnostic markers of AD. The authors also demonstrated the role of *PMAIP1* in AD and its impact on mitochondrial function using cellular experiments, which provides a potential therapeutic target for AD.

## CRediT authorship contribution statement

**Yingchun Ling:** Writing – original draft. **Lingmin Hu:** Validation. **Jie Chen:** Methodology. **Mingyong Zhao:** Software. **Xinyang Dai:** Conceptualization, Funding acquisition, Resources, Writing – review & editing, Supervision.

## Conflicts of interest

The authors declare that they have no known competing financial interests or personal relationships that could have appeared to influence the work reported in this paper.

## Appendix Supplementary materials

Image, application 1
