# Supplementary material for: The mechanism of mitochondrial metabolic gene PMAIP1 involved in Alzheimer's disease process based on bioinformatics analysis and experimental validation
Source: Clinics (Sao Paulo). 2024 Apr 30;79:100373. doi: 10.1016/j.clinsp.2024.100373 (PMC11070595; doi:10.1016/j.clinsp.2024.100373)

**CLINICS-D-23-00699_Supplementary Material**

**Supplementary Material 1** Western blots of GAPDH, *PMAIP1*, BCL2, Bax, cleaved caspase3.

**Figure 7** B-GAPDH **Figure 7** B-PMAIP1


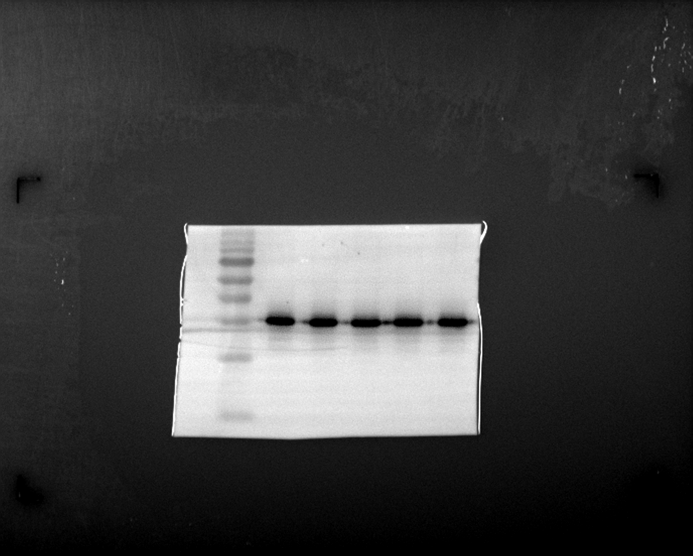

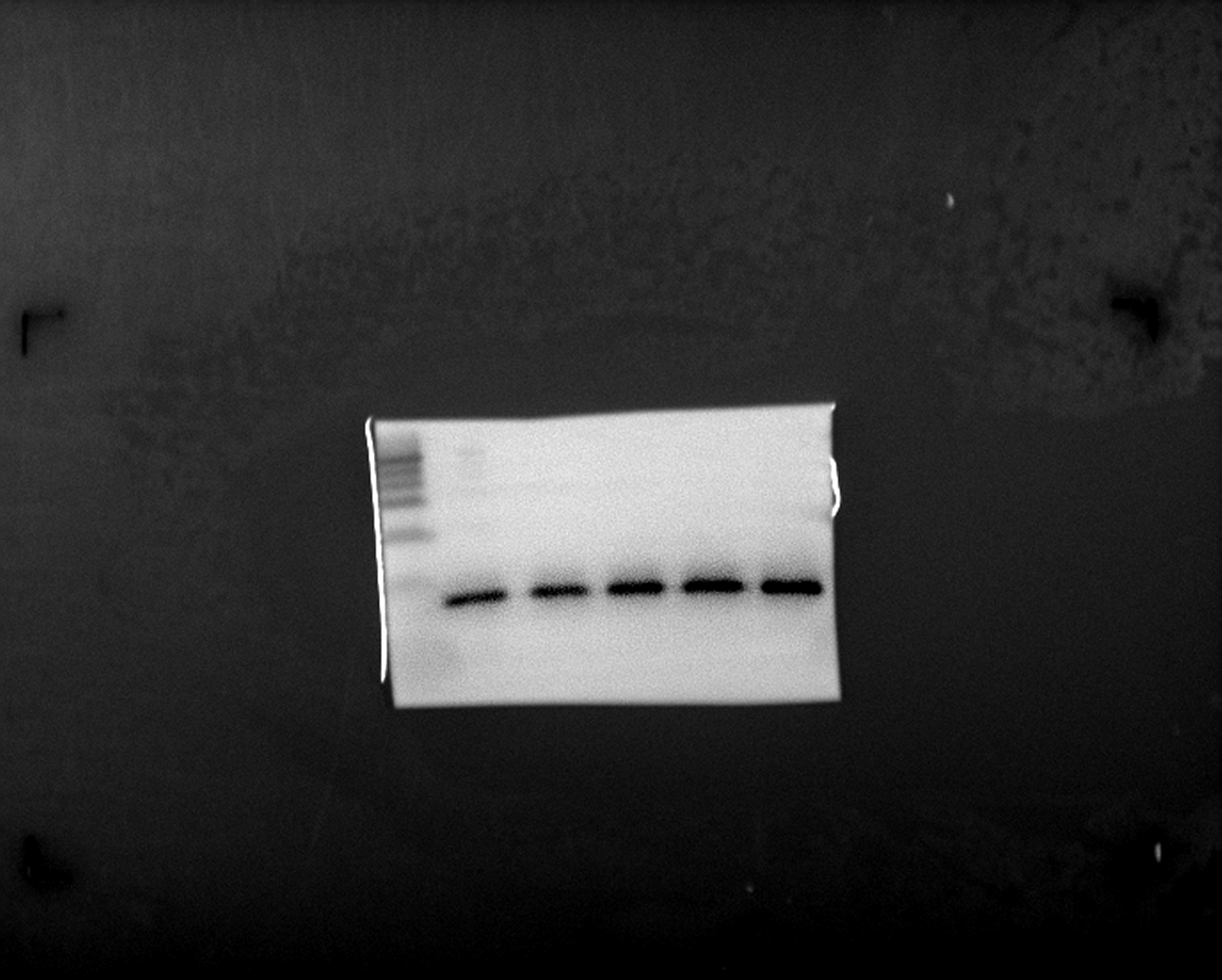


**Figure 8** B-GAPDH **Figure 8** B-PMAIP1


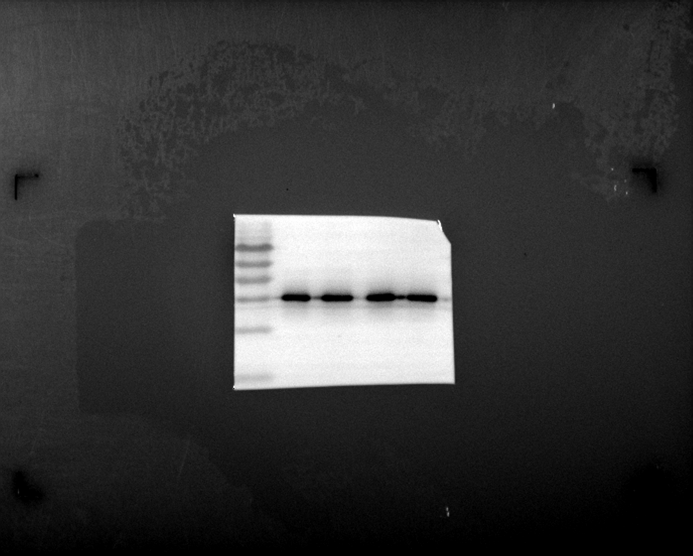

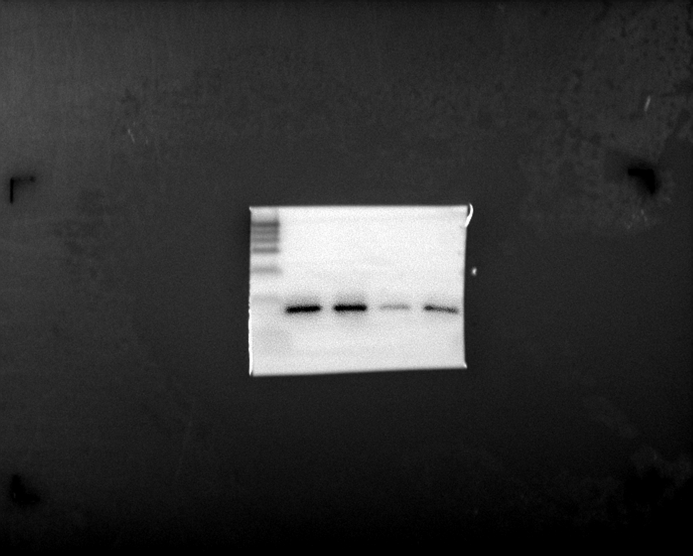


**Figure 8** D-GAPDH **Figure 8** D-cleaved caspase3


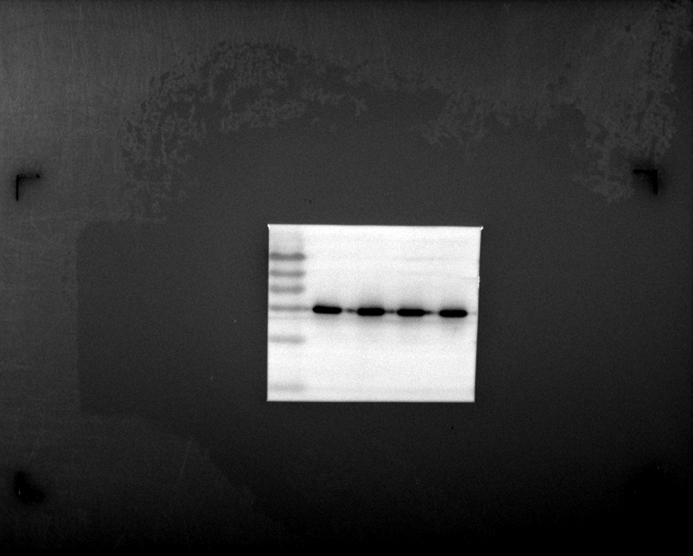

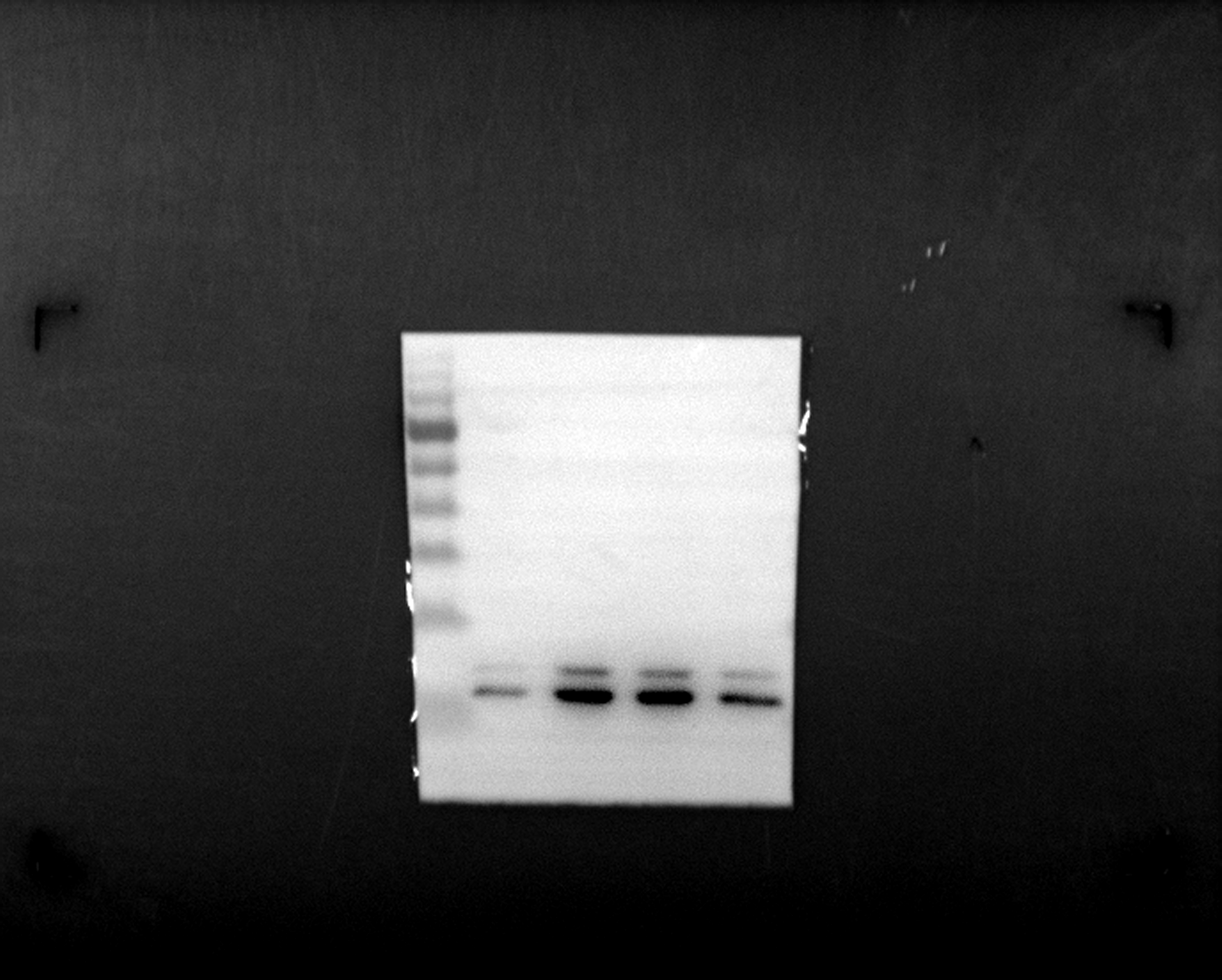


**Figure 8** D-Bax **Figure 8** D-BCL2


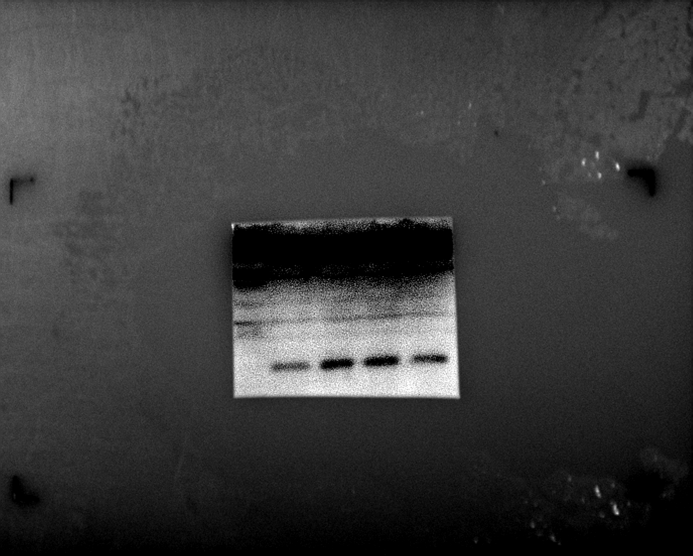

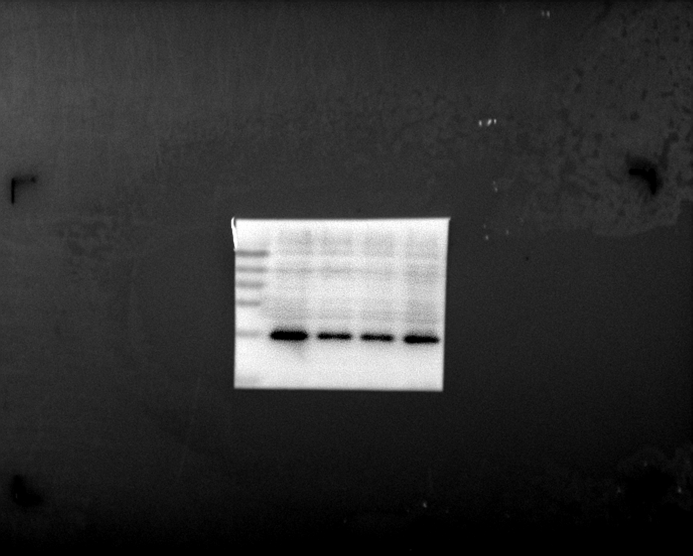

Supplement: Supplementary file 1 [file mmc1.docx]
